# Altered Endocannabinoid Signaling in Placentas from SARS-CoV-2-Infected Pregnancies

**DOI:** 10.3390/diagnostics16050690

**Published:** 2026-02-26

**Authors:** Özge Kaplan, Mehmet Uğur Karabat, Süreyya Özdemir Başaran, Dilek Yavuz, Fırat Aşır, Tuğcan Korak, Elif Ağaçayak, Engin Deveci

**Affiliations:** 1 Department of Histology and Embryology, Medical Faculty, Dicle University, 21280 Diyarbakır, Turkeyfiratasir@gmail.com (F.A.); 2 Department of Andrology, Gazi Yasargil Education and Research Hospital, Health Science University, 21090 Diyarbakır, Turkey; 3 Department of Medical Biology, Medical Faculty, Kocaeli University, 41380 Kocaeli, Turkey; 4 Department of Gynecology and Obstetrics, Medical Faculty, Dicle University, 21280 Diyarbakır, Turkey

**Keywords:** COVID-19, placenta, cannabinoid receptors, endocannabinoid system, histopathology, digital pathology

## Abstract

**Background:** SARS-CoV-2 infection during pregnancy has been associated with systemic inflammatory responses and placental pathology; however, the molecular mechanisms underlying placental involvement remain incompletely understood. The endocannabinoid system plays a critical role in placental development, immune regulation, and vascular homeostasis. **Materials and Methods:** Placental tissues were obtained from 20 healthy pregnant women and 20 women with confirmed SARS-CoV-2 infection who had recovered by the time of delivery. Demographic and laboratory parameters were recorded. Histopathological evaluation was performed using hematoxylin and eosin staining. Immunohistochemical analysis of cannabinoid receptor 1 (CNR1) and cannabinoid receptor 2 (CNR2) expression was conducted, supported by quantitative digital image analysis using QuPath. Network-based protein–protein interaction and Kyoto Encyclopedia of Genes and Genomes (KEGG) pathway enrichment analyses were performed to explore potential molecular mechanisms. **Results:** COVID-19-positive placentas exhibited prominent histopathological alterations, including increased fibrinoid deposition, syncytial knot formation, vascular congestion, and intervillous inflammatory cell infiltration. Systemic inflammatory and coagulation markers, particularly neutrophil percentage, C-reactive protein, D-dimer, and fibrinogen levels, were significantly elevated in the COVID-19 group. CNR1 and CNR2 expressions were markedly increased across multiple placental compartments, including decidual cells, trophoblastic layers, syncytial knots, and Hofbauer cells. Quantitative digital analysis confirmed significant upregulation of both receptors. Bioinformatic analysis revealed enrichment of endocannabinoid signaling, cAMP-related pathways, and inflammatory mediator regulation of TRP channels. **Conclusions:** The findings indicate that SARS-CoV-2 infection is associated with coordinated inflammatory, structural, and molecular alterations in the placenta. Upregulation of CB1 and CB2 suggests an active involvement of the endocannabinoid system in placental immune and vascular responses to COVID-19, highlighting its potential relevance for understanding placental pathology associated with maternal viral infections

## 1. Introduction

Viral infections during pregnancy represent a significant clinical challenge due to their potential to disrupt maternal–fetal homeostasis and compromise placental function [1,2]. The placenta acts not only as a physical barrier but also as a dynamic immunological interface that regulates inflammatory responses, vascular remodeling, and nutrient exchange between the mother and fetus [3]. Viral entry into placental tissues may occur via hematogenous spread or ascending infection from the lower genital tract, with the extent of placental involvement largely determined by viral tropism, receptor expression, and host immune responses [4]. Even without direct fetal infection, placental inflammation and vascular injury may adversely affect pregnancy outcomes [5,6].

Since its emergence in late 2019, severe acute respiratory syndrome coronavirus 2 (SARS-CoV-2) has posed unique risks to pregnant women [7]. Accumulating evidence indicates that COVID-19 is associated with increased maternal morbidity, heightened systemic inflammation, and coagulation abnormalities, which may persist even after clinical recovery [8,9]. Importantly, several studies have demonstrated characteristic placental alterations in SARS-CoV-2-positive pregnancies, including fibrin deposition, syncytial knot formation, intervillous inflammation, and vascular congestion, suggesting that placental pathology may reflect systemic maternal immune dysregulation rather than direct viral cytopathic effects alone [10,11].

The ECS, composed primarily of CNR1, CNR2 and their endogenous ligands, plays a crucial regulatory role in female reproduction. ECS signaling is involved in implantation, decidualization, trophoblast differentiation, angiogenesis, and immune modulation at the maternal–fetal interface [12,13]. CNR1 is predominantly associated with cellular differentiation, vascular tone, and apoptosis, whereas CNR2 is mainly expressed in immune cells and is implicated in the regulation of inflammatory and immunosuppressive responses [13,14]. Dysregulation of ECS signaling has been linked to adverse pregnancy conditions such as preeclampsia, fetal growth restriction, and placental insufficiency [15].

Recent studies have suggested that viral infections and systemic inflammatory states may alter ECS activity, potentially amplifying local immune responses and contributing to tissue injury [16]. However, data regarding the involvement of cannabinoid receptors in placental pathology associated with COVID-19 remain limited. In particular, the relationship between systemic inflammatory and coagulation disturbances observed in COVID-19 and localized placental ECS alterations has not been comprehensively explored [17].

Given the pronounced inflammatory, vascular, and histopathological changes reported in COVID-19 placentas, we hypothesized that SARS-CoV-2 infection is associated with dysregulated CNR1 and CNR2 expression across multiple placental compartments. Furthermore, we postulated that these alterations may be linked to key inflammatory and signaling pathways involved in immune activation and vascular remodeling. Therefore, the present study aimed to investigate CNR1 and CNR2 expression patterns in placentas from COVID-19-positive pregnancies without additional obstetric complications to correlate these findings with systemic laboratory markers and histopathological alterations, and to explore potential molecular mechanisms through network-based pathway enrichment analysis.

## 2. Materials and Methods

### 2.1. Study Design and Ethical Approval

This cross-sectional case–control study was conducted at Dicle University Faculty of Medicine. Ethical approval was obtained from the Non-Interventional Clinical Research Ethics Committee of Dicle University (15 February 2022, No: 39) and the Turkish Ministry of Health (10 January 2022, Record No: T09_45_26). The study was conducted according to the principles of the Declaration of Helsinki. Written informed consent was obtained from all participants prior to enrollment.

### 2.2. Study Population and Clinical Data Collection

A total of 40 pregnant women aged between 18 and 49 years were included in the study. The study group consisted of 20 women who were diagnosed with SARS-CoV-2 infection during pregnancy and had recovered by the time of delivery, confirmed by real-time polymerase chain reaction (RT-PCR). All SARS-CoV-2 infections in the study group occurred during the second and mostly third trimester of pregnancy. The control group included 20 healthy pregnant women with no history of COVID-19 infection. Patients with additional pregnancy-related complications, including preeclampsia, gestational diabetes mellitus, fetal growth restriction, placental abnormalities, or chronic systemic diseases, were excluded to minimize confounding factors. Maternal demographic characteristics, obstetric data, and laboratory parameters were obtained from hospital medical records.

### 2.3. Laboratory Parameters

Laboratory data collected at the time of COVID-19 diagnosis included complete blood count parameters (white blood cell count, neutrophil and lymphocyte counts and percentages, hemoglobin, hematocrit, platelet count), inflammatory markers (C-reactive protein, ferritin, procalcitonin), and coagulation parameters (D-dimer, fibrinogen, international normalized ratio). These parameters were used to assess systemic inflammatory and coagulation status and to correlate clinical findings with placental histopathological and immunohistochemical alterations. Ferritin levels were assessed as part of routine inflammatory evaluation, as ferritin is an acute-phase reactant frequently elevated in SARS-CoV-2 infection and associated with systemic inflammatory response. In the COVID-19 group, laboratory parameters were obtained on the day of SARS-CoV-2 diagnosis as part of routine clinical evaluation. In the control group, laboratory data were collected at the time of delivery during routine obstetric assessment.

### 2.4. Placental Tissue Collection

Placental tissue samples were obtained immediately after delivery. Representative samples were taken from the maternal side of the placenta, including decidual tissue and chorionic villi, avoiding areas of macroscopic infarction or calcification. For each placenta, two representative tissue blocks were obtained from the maternal side, including decidual tissue and chorionic villi. From each paraffin block, three serial sections (5 μm thickness) were prepared for histological and immunohistochemical analyses. Sampling was standardized across all cases to ensure consistency between groups. Tissue samples were fixed in zinc formalin for routine histological and immunohistochemical analysis.

### 2.5. Histological Tissue Processing and Hematoxylin and Eosin Staining

Fixed placental tissues were processed through a graded alcohol series, cleared in xylene, and embedded in paraffin wax. Serial sections of 5 μm thickness were cut using a microtome (Leica RM2265, Leica Biosystems, Wetzlar, Germany). Sections were stained with Mayer’s hematoxylin solution (catalog no: MHS16, Sigma-Aldrich, St. Louis, MO, USA) and alcoholic eosin Y solution (catalog no: HT110132, Sigma-Aldrich, St. Louis, MO, USA) for routine histopathological evaluation and evaluation of general placental morphology. Histopathological assessment focused on villous architecture, fibrinoid deposition, syncytial knot formation, inflammatory cell infiltration, decidual nuclear morphology, vascular dilatation, congestion, and basement membrane thickness.

### 2.6. Immunohistochemical Analysis

Immunohistochemical staining was performed to assess CNR1 and CNR2 expression. Paraffin sections were deparaffinized, rehydrated through descending ethanol concentrations, and rinsed in distilled water. Endogenous peroxidase activity was blocked using hydrogen peroxide solution (TA-015-HP, Thermo Fisher Scientific, Fremont, CA, USA) for 20 min. Antigen retrieval was performed using EDTA buffer (pH:8.0, catalog no: ab93680, Abcam, Cambridge, MA, USA) in a microwave oven for 10 min. After cooling to room temperature, sections were rinsed in phosphate-buffered saline and processed for subsequent immunohistochemical steps. After blocking, sections were incubated overnight at 4 °C with primary antibodies against CNR1 (ab23703, Abcam, USA, dilution ratio: 1/100) and CNR2 (ab3561, Abcam, USA, dilution ratio: 1/100). After primary antibody incubation, sections were incubated with a biotinylated secondary antibody (catalog no: TP-015-BN, Thermo Fisher Scientific, Fremont, CA, USA), followed by visualization with DAB chromogen (DAB; TA-001-HCX, Thermo Fisher Scientific, Fremont, CA, USA). Sections were counterstained with Harris hematoxylin, mounted, and examined using a Zeiss Imager A2 light microscope (Jena, Germany).

### 2.7. Evaluation of Immunoreactivity

Quantitative evaluation of CNR1 and CNR2 immunoreactivity was performed using the open-source digital pathology software QuPath (version 0.5.1). Whole-slide images were acquired using a Zeiss Imager A2 light microscope under standardized illumination and magnification settings and imported into QuPath for analysis. For each placental sample, five representative regions of interest (ROIs) were manually selected from well-preserved areas of the decidua and chorionic villi, avoiding tissue folds, necrotic regions, hemorrhage, or staining artifacts. The same number of ROIs was analyzed per case to ensure consistency between groups. Placental compartments evaluated included decidual cells, cytotrophoblasts, syncytiotrophoblasts, syncytial knots, Hofbauer cells, and villous connective tissue. Color deconvolution was applied to separate hematoxylin and DAB signals using the built-in QuPath stain vector estimation. Cell detection was performed using the “Positive Cell Detection” algorithm, with optimized threshold parameters applied uniformly across all slides. DAB optical density thresholds were defined to classify cells as negative or positive, and staining intensity was categorized as weak, moderate, or strong based on optical density values. For each ROI, the following quantitative parameters were recorded: Total cell count, Number and percentage of CNR1- or CNR2-positive cells, and Mean DAB optical density. H score was formulated using these parameters. All digital analyses were performed by an investigator blinded to the clinical grouping. The obtained quantitative data were used for statistical comparison between the control and COVID-19 groups and to support the semiquantitative immunohistochemical observations [18].

### 2.8. Protein–Protein Interaction Network and Pathway Enrichment Analysis

To explore potential molecular mechanisms associated with CNR1 and CNR2 upregulation, protein–protein interaction (PPI) network analysis was performed using Cytoscape software (version 3.10.1). The STRING database was utilized to identify interacting proteins of CNR1 and CNR2, with a confidence score threshold of ≥0.4. KEGG pathway enrichment analysis was conducted, and pathways with a false discovery rate (FDR) < 0.05 were considered significant. The top enriched pathways were visualized as ring charts integrated into the PPI network [19].

### 2.9. Statistical Analysis

Statistical analyses were performed using IBM SPSS Statistics software version 25 (IBM Corp., Armonk, NY, USA). Clinical continuous variables are expressed as medians (minimum–maximum), and categorical variables are presented as counts and percentages. The Shapiro–Wilk test was used to assess data distribution. Between-group comparisons were conducted using the Mann–Whitney U test for non-normally distributed continuous variables and the chi-square test for categorical variables. A *p* value < 0.05 was considered statistically significant. Quantitative QuPath-derived variables, including percentage of positive cells, mean optical density, and H-scores, were included in the statistical analysis. Between-group comparisons were performed using the Mann–Whitney U test. Correlations between QuPath-derived CNR1/CNR2 expression levels and systemic inflammatory or coagulation parameters (CRP, neutrophil percentage, D-dimer, fibrinogen) were explored using Spearman correlation analysis. A *p* value < 0.05 was considered statistically significant.

## 3. Results

### 3.1. COVID-19 Significantly Increased Neutrophil, CRP, D-Dimer and Fibrinogen

Table 1 summarizes the demographic and obstetric characteristics of the control (COVID−) and COVID-19-positive groups. Maternal age, gravida, and parity values were comparable between the two groups, with no statistically significant differences observed (*p* > 0.05 for all). The mode of delivery did not differ significantly between groups; normal vaginal delivery was the predominant mode in both cohorts. These findings indicate that the study groups were demographically and obstetrically well matched, minimizing potential confounding effects related to baseline maternal characteristics.

As shown in Table 2, several laboratory parameters differed significantly between the control and COVID-19 groups. The percentage of neutrophils was significantly higher in the COVID-19 group compared with controls (*p* = 0.036), whereas lymphocyte percentages did not differ significantly. Inflammatory markers, including C-reactive protein, were markedly elevated in the COVID-19 group (*p* = 0.021). In addition, coagulation-related parameters demonstrated significant alterations, with both D-dimer and fibrinogen levels being significantly increased in COVID-19-positive patients (*p* = 0.001 and *p* = 0.005, respectively). Other hematological and biochemical parameters, including hemoglobin, platelet count, liver enzymes, procalcitonin, and INR, did not show statistically significant differences between groups. The majority of patients in the COVID-19 group presented with mild to moderate disease, and no severe cases requiring intensive care were included. Procalcitonin levels were assessed as part of routine inflammatory evaluation to exclude possible bacterial superinfection and to further characterize systemic inflammatory status.

### 3.2. COVID-19 Adversely Altered Histology of Placenta

**Figure 1:**
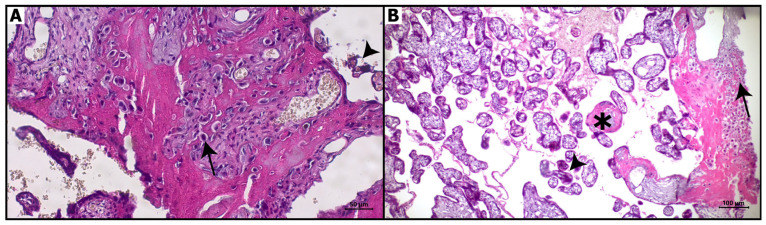
Hematoxylin and eosin staining of placental sections. (**A**): Control group: Normal appearance of decidual cells (arrow) and chorionic villi (arrowhead), Bar: 50 μm (magnification: 20×). (**B**): COVID-19 group: Pyknotic nuclei of decidua cells (arrow), fibrinoid deposits (asterisk), and syncytial knots (arrowhead). Bar: 100 μm (magnification: 10×).

Hematoxylin and eosin staining of the control and COVID-19 groups is shown in Figure 1. In the control group, normal placental histology was observed. The placental villi were regular and contained fetal capillaries (Figure 1A). In the COVID-19 group, pyknosis and degenerative changes were observed in the nuclei of decidual cells. Fibrinoid deposition in the chorionic villi increased, and the number of syncytial knots was elevated. Leukocyte infiltration was present in the intervillous areas. In addition, vascular dilatation and congestion were observed in the fetal capillaries (Figure 1B).

Table 3 presents the histopathological characteristics of placental tissues from the control and COVID-19 groups. Placentas from the control group demonstrated preserved villous architecture with minimal fibrinoid deposition, rare syncytial knots, and absence of inflammatory cell infiltration. In contrast, placentas from the COVID-19 group exhibited prominent structural alterations, including disruption of normal villous architecture, increased fibrinoid deposition, and a higher number of syncytial knots (Table 3). Additionally, decidual cells in the COVID-19 group showed nuclear pyknosis, accompanied by evident leukocyte infiltration in the intervillous spaces. Fetal capillaries displayed dilatation and congestion, and thinning of the villous basement membrane was observed in COVID-19 placentas.

### 3.3. CNR1 and CNR2 Expressions Were Upregulated After COVID-19 Infection

**Figure 2:**
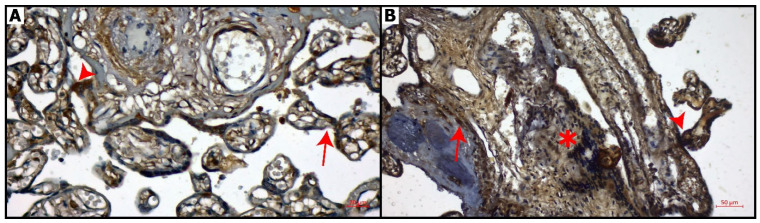
CNR1 immunostaining of placental sections. (**A**): Control group: Weak expression of CNR1in chorionic villi (arrow), syncytial knots (arrowhead), Bar: 20 μm (magnification: 40×), (**B**): COVID-19 group: Intense expression of CNR1 in decidual cells (arrow), syncytial knots (arrowhead) and connective tissue (asterisk). Bar: 50 μm (magnification: 20×).

Placental sections immunostained with CB-1 are shown in Figure 2. Control group: CNR1 expression was predominantly negative in chorionic villi. CNR1 expression was positive in decidua cells (Figure 2A). COVID-19 group: CNR1 expression was increased in decidual cells, chorionic villi, and syncytial knots compared to control group (Figure 2B).

**Figure 3:**
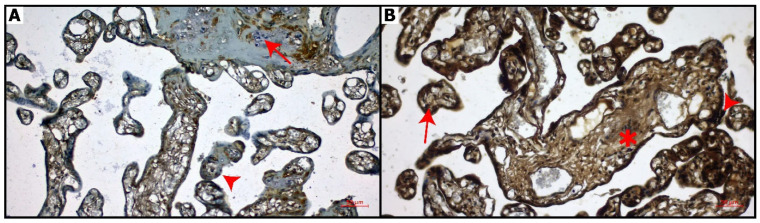
CNR2 immunostaining of placental sections. (**A**): Control group: Weak expression of CNR2 in decidual cells (arrow) and chorionic villi (arrowhead), (**B**): COVID-19 group: Intense expression of CNR2 in chorionic villi (arrow), syncytial knots (arrowhead) and connective tissue (asterisk). Bar: 50 μm (magnification: 20×).

Placental sections immunostained with CB-2 are shown in Figure 3. Control group: CNR2 expression was mainly negative in chorionic villi and decidual cells (Figure 3A). COVID-19 group: CNR1 was highly expressed in decidual cells, chorionic villi, and syncytial knots compared to the control group (Figure 3B).

The distribution of CNR1 and CNR2 immunoreactivity in placental compartments is summarized in Table 4. In the control group, CNR1 expression was limited, with weak or absent immunoreactivity observed in trophoblastic layers and stromal components. In contrast, placentas from the COVID-19 group demonstrated markedly increased CNR1 expression. Strong CNR1 immunoreactivity was detected in decidual cells, while cytotrophoblasts and syncytiotrophoblasts showed clear positive staining. Syncytial knots exhibited intense CNR1 expression, and Hofbauer cells and villous connective tissue displayed positive immunoreactivity in the COVID-19 group. CNR2 immunoreactivity was minimal or absent in most placental compartments of the control group. In contrast, placentas from COVID-19-positive patients exhibited a pronounced increase in CNR2 expression. Strong CNR2 immunoreactivity was observed in decidual cells, cytotrophoblasts, and syncytiotrophoblasts. Syncytial knots demonstrated intense CNR2 staining, and positive immunoreactivity was also noted in Hofbauer cells and villous connective tissue. These findings indicate an upregulation of CNR2 expression in immune-related and trophoblastic compartments of the placenta following COVID-19 infection.

Quantitative digital image analysis using QuPath demonstrated a significant increase in both CNR1 and CNR2 expression in placentas from COVID-19-positive patients compared with controls (Table 5). Median CNR1 H-score values were markedly higher in the COVID-19 group, indicating widespread upregulation across trophoblastic and decidual compartments (*p* < 0.001). Similarly, CNR2 H-scores showed a pronounced increase in COVID-19 placentas, particularly in immune-related cell populations (*p* < 0.001). When CNR1 and CNR2 H-scores were combined, the difference between groups became even more prominent, supporting the potential diagnostic value of combined cannabinoid receptor expression.

Table 6 provides an integrated summary of the key diagnostic-relevant findings observed in COVID-19 placentas. Despite the absence of additional pregnancy-related complications, COVID-19-positive patients demonstrated significant systemic inflammatory and coagulation changes, as evidenced by elevated CRP, neutrophil percentage, D-dimer, and fibrinogen levels. These systemic alterations were accompanied by distinct placental morphological changes, including increased fibrinoid deposition, syncytial knot formation, and inflammatory cell infiltration. Concurrently, both CNR1 and CNR2 expressions were markedly increased across multiple placental compartments, highlighting a consistent immunohistochemical response associated with COVID-19 infection.

### 3.4. PPI Network and KEGG Pathway Enrichment Analysis

**Figure 4:**
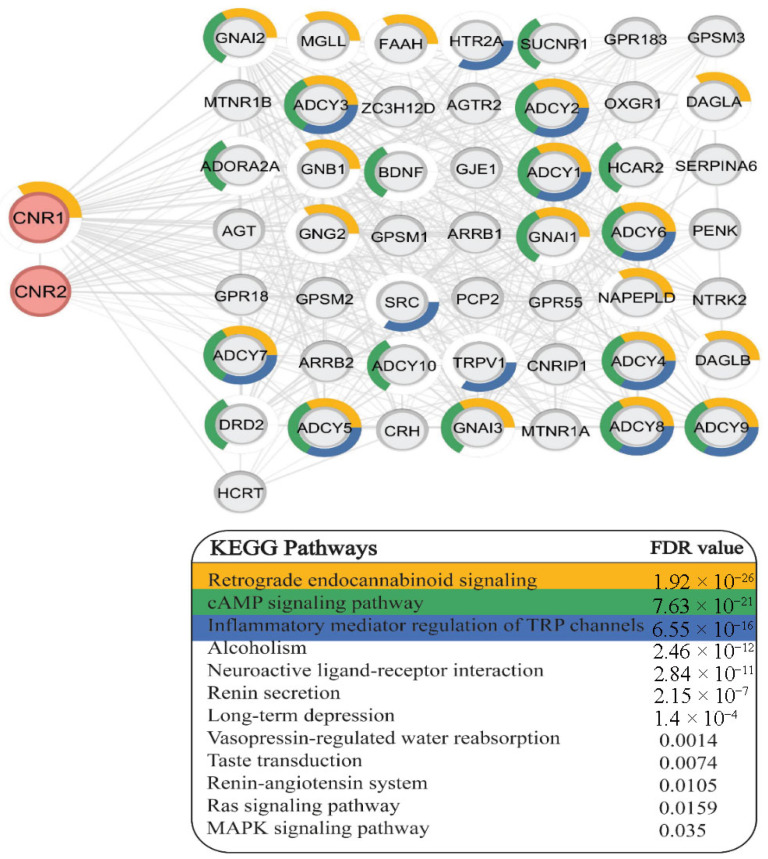
Network-based KEGG pathway enrichment analysis. The shared interactors of CNR1 and CNR2 were significantly enriched in 12 KEGG pathways, ranked according to their FDR values. The first three were represented as ring charts surrounding the correlated nodes.

After conducting CNR1- and CNR2-associated network analysis, the PPI network comprising 52 nodes and 469 edges was obtained. The KEGG database revealed 12 enriched pathways correlated with both target genes. “Retrograde endocannabinoid signaling”, “cAMP signaling pathway” and “Inflammatory mediator regulation of transient receptor potential (TRP) channels” were most likely to be significantly associated with CNR1 and CNR2, and they were represented with yellow, green and blue colors, respectively. The nodes in the PPI network associated with these annotations were displayed within a colored ring chart. Remarkably, the adenylate cyclase (ADCY) 1–9 involved in the PPI network of the target genes was found to be linked with all top three enriched pathways (Figure 4).

## 4. Discussion

This study demonstrates that SARS-CoV-2 infection during pregnancy is associated with pronounced systemic inflammatory and coagulation alterations that are mirrored by distinct histopathological and molecular changes in placental tissue. Beyond descriptive histology, our findings highlight a consistent upregulation of CNR1 and CNR2 across multiple placental compartments and provide quantitative evidence supporting their potential diagnostic relevance in COVID-19-affected pregnancies [20,21].

Systemic inflammation and coagulation dysregulation are well-established hallmarks of COVID-19 [22]. Elevated neutrophil percentages, CRP, D-dimer, and fibrinogen levels observed in the present cohort indicate a sustained inflammatory and prothrombotic state, even in pregnancies without additional obstetric complications [23,24]. The placenta, as a highly vascularized and immunologically active organ, appears particularly susceptible to these systemic perturbations. Increased fibrinoid deposition, syncytial knot formation, vascular congestion, and intervillous leukocyte infiltration observed in COVID-19 placentas are consistent with maternal vascular malperfusion and inflammatory placentitis reported in previous studies [25,26,27]. These alterations suggest impaired placental perfusion and adaptive trophoblastic responses to inflammatory stress rather than direct viral cytopathic damage alone.

A key novel aspect of this study is the demonstration of marked CNR1 and CNR2 upregulation in COVID-19 placentas, supported by both semiquantitative immunohistochemistry and objective digital image analysis [28]. CNR1 expression was predominantly increased in decidual cells and trophoblastic layers, structures critical for placental anchoring, vascular remodeling, and barrier integrity [14,29]. Given the established role of CNR1 signaling in apoptosis, cAMP modulation, and trophoblast differentiation, its overexpression may contribute to the increased syncytial knot formation and nuclear pyknosis observed histologically [30,31]. These findings align with previous reports linking CNR1 activation to impaired decidualization and placental insufficiency in pathological pregnancies.

CNR2 upregulation was particularly prominent in immune-related placental compartments, including Hofbauer cells and intervillous inflammatory infiltrates [32]. CNR2 is primarily associated with immunomodulatory and anti-inflammatory signaling in peripheral immune cells [33]. Its increased expression in COVID-19 placentas may reflect a compensatory response aimed at limiting excessive inflammation at the maternal–fetal interface [34]. However, dysregulated or prolonged CNR2 activation may also alter macrophage polarization, cytokine release, and trophoblast–immune cell crosstalk, potentially exacerbating placental dysfunction under sustained inflammatory conditions [35].

Importantly, integration of QuPath-derived quantitative data enabled evaluation of the diagnostic performance of CNR1 and CNR2 expression. Both receptors demonstrated good discriminative ability in differentiating COVID-19-positive placentas from controls, with further improvement observed when CNR1 and CNR2 H-scores were combined. These findings suggest that placental cannabinoid receptor expression reflects a reproducible molecular signature associated with maternal SARS-CoV-2 infection. From a diagnostic perspective, such markers may serve as objective indicators of placental involvement in COVID-19, complementing conventional histopathological assessment and systemic laboratory parameters.

Bioinformatic analysis further supports the biological plausibility of these observations. Enrichment of endocannabinoid signaling, cAMP pathways, and TRP channel regulation underscores the intersection between cannabinoid receptor activation, inflammatory signaling, and vascular responses. The strong association of CNR1 and CNR2 with adenylate cyclase isoforms suggests that altered cAMP dynamics may represent a central mechanism linking ECS dysregulation to trophoblastic apoptosis and immune modulation. Moreover, the involvement of TRP channels, known regulators of immune cell activation and cytokine release, provides an additional mechanistic layer through which ECS activation may influence placental inflammation in COVID-19.

Several limitations should be acknowledged. The relatively small sample size and single-center design may limit generalizability. In addition, placental tissue was analyzed at delivery, precluding assessment of temporal changes in cannabinoid receptor expression during different stages of infection. Functional assays were not performed to directly confirm downstream signaling effects of CNR1 and CNR2 activation. Nevertheless, the integration of histopathology, digital quantification, ROC-based diagnostic evaluation, and bioinformatic analysis represents a comprehensive approach that strengthens the translational relevance of our findings.

## 5. Conclusions

This study demonstrates that SARS-CoV-2 infection during pregnancy is associated with distinct placental alterations characterized by inflammatory, vascular, and structural changes, even in the absence of additional obstetric complications. The consistent upregulation of CNR1 and CNR2 across multiple placental compartments suggests that the endocannabinoid system is actively involved in the placental response to maternal COVID-19. Beyond conventional histopathological findings, the increased expression of CNR1 and CNR2 reflects a reproducible molecular pattern linked to systemic inflammation and immune dysregulation. These receptors appear to integrate signals related to trophoblastic integrity, immune cell activation, and vascular remodeling at the maternal–fetal interface. From a diagnostic perspective, assessment of placental cannabinoid receptor expression may provide complementary information to routine histopathology by highlighting underlying molecular responses to maternal SARS-CoV-2 infection. Although further studies are required to validate these findings in larger cohorts and to clarify their temporal and functional implications, our results suggest that CNR1 and CNR2 expression patterns may represent informative tissue-based markers of placental involvement in COVID-19. This approach may contribute to improved characterization of placental pathology associated with maternal viral infections and enhance the interpretative value of placental examination in clinical practice.

## Figures and Tables

**Table 1:** Demographic characteristics of the patient and control groups.

*n* = 20		Control	COVID-19	*p* Value
Age (years), median (min-max)		27 (20–42)	32 (24–48)	ns
Gravida, median (min-max)		2 (1–4)	4 (2–9)	ns
Parity, median (min-max)		2 (0–3)	4 (2–7)	ns
Normal Vaginal Birth, n (percentage)		19 (%95)	15 (%75)	ns
Cesarean Section, n (percentage)		1 (%5)	5 (%25)	ns
Gestational trimester at infection	Second		8 (%40)	-
	Third		12 (%60)	

ns: non-significant, Statistical note: Continuous variables: Mann–Whitney U test. Categorical variables: Chi-square test. ns: non-significant.

**Table 2:** Laboratory parameters per control and COVID-19 groups.

*n* = 20	Control	COVID-19	Significance
WBC	9.5 (3.4–12.1)	10.4 (5–14.8)	0.144
NEU (%)	65.4 (42.5–87.7)	77.15 (45.5–91.5)	**0.036**
LYM (%)	21.2 (6.5–46.3)	16.30 (4.2–45)	0.159
NEU	7.5 (2.4–8.6)	7.65 (0–13.2)	0.588
LYM	1.6 (0.6–3)	1.5 (0.4–8.7)	0.542
HGB	11.4 (9.1–13.1)	11.7 (9–14)	0.499
HCT (%)	33.2 (25.8–39.7)	34.40 (25.9–42)	0.552
PLT	220 (144–385)	239 (138–400)	0.317
AST	24.0 (10–56)	29.50 (13–194)	0.323
ALT	19.5 (7–49)	18.00 (7–231)	0.828
CRP	4.4 (1.2–57.1)	17.80 (1.2–176.7)	**0.021**
FERRITIN	25.4 (5.9–65.1)	43.70 (6.1–97.5)	0.256
D DIMER	245.0 (140–2630)	1070.0 (150–5080)	**0.001**
FIBRINOGEN	208.5 (175.56–395.6)	412.7 (180–644.23)	**0.005**
PCT	0.05 (0.02–0.18)	0.05 (0.01–1.09)	0.724
INR	0.98 (0.79–1.19)	0.96 (0.85–1.08)	0.272

Data was shown as median (min–max). WBC: White blood cell; NEU: Neutrophil; LYM: Lymphocyte; HGB: Hemoglobin; HCT: Hematocrit; PLT: Platelet; AST: Aspartate aminotransferase; ALT: Alanine aminotransferase; CRP: C-reactive protein; PCT: Procalcitonin; INR: International Normalized Rate. Bold formatting is used to indicate statistical significance (*p* < 0.005).

**Table 3:** Histopathological Findings in Placenta.

Histopathological Feature	Control (COVID−)	COVID-19 (+)
Normal villous architecture	Preserved	Disrupted
Decidual nuclear pyknosis	Absent	Present
Fibrinoid deposition	Minimal	Increased
Syncytial knots	Rare	Increased
Intervillous leukocyte infiltration	Absent	Present
Fetal capillary dilatation/congestion	Absent	Present
Basement membrane thickness	Normal	Thinned

**Table 4:** Distribution of CNR1 and CNR2 immunoreactivity.

	CNR1 Expression		CNR2 Expression	
Placental Compartment	Control (COVID−)	COVID-19 (+)	Control (COVID−)	COVID-19 (+)
Decidual cells	Positive	Strongly positive	Negative	Strongly positive
Cytotrophoblast	Negative	Positive	Negative	Positive
Syncytiotrophoblast	Negative	Positive	Negative	Positive
Syncytial knots	Weak	Strong	Weak	Strong
Hofbauer cells	Negative	Positive	Negative	Positive
Villous connective tissue	Weak	Positive	Weak	Positive

**Table 5:** Quantitative QuPath Analysis of CNR1 and CNR2 Expression (H-score).

Marker	Group	H-Score (Median, Min–Max)	*p* Value
CNR1	Control (COVID−)	45 (20–80)	<0.001
	COVID-19 (+)	165 (110–240)	
CNR2	Control (COVID−)	30 (10–65)	<0.001
	COVID-19 (+)	185 (120–260)	
CNR1 + CNR2 (combined)	Control (COVID−)	75 (40–130)	<0.001
	COVID-19 (+)	350 (250–480)	

Statistical note: Data are presented as median (minimum–maximum). Between-group comparisons were performed using the Mann–Whitney U test.

**Table 6:** Summary of Findings in COVID-19 Placentas.

Category	Key Finding
Inflammatory markers	CRP, neutrophil %, fibrinogen significantly increased
Coagulation profile	D-dimer and fibrinogen significantly elevated
Placental morphology	Increased fibrinoid deposition, syncytial knots, leukocyte infiltration
CNR1 expression	Markedly increased in trophoblasts and decidua
CNR2 expression	Markedly increased in immune-related placental cells
Pregnancy complications	None present

## Data Availability

The datasets used and/or analyzed during the current study are available from the corresponding author on reasonable request.
